# Intracerebral Hemorrhage in COVID-19 Patients with Pulmonary Failure: A Propensity Score-Matched Registry Study

**DOI:** 10.1007/s12028-021-01202-7

**Published:** 2021-02-23

**Authors:** Corinna N. Lang, Johanna S. Dettinger, Michael Berchtold-Herz, Stefan Utzolino, Xavier Bemtgen, Viviane Zotzmann, Bonaventura Schmid, Paul M. Biever, Christoph Bode, Katharina Müller-Peltzer, Daniel Duerschmied, Tobias Wengenmayer, Wolf-Dirk Niesen, Dawid L. Staudacher

**Affiliations:** 1 Department of Cardiology and Angiology I, Faculty of Medicine, Heart Center Freiburg University, University of Freiburg, Hugstetter Str. 55, 79106 Freiburg, Germany; 2 Department of Medicine III (Interdisciplinary Medical Intensive Care), Medical Center, Faculty of Medicine, University of Freiburg, Freiburg, Germany; 3 Department of Cardiovascular Surgery, Faculty of Medicine, Heart Center Freiburg University, University of Freiburg, Freiburg, Germany; 4 Department of General and Visceral Surgery, Medical Center, University of Freiburg, Freiburg, Germany; 5 Department of Emergency Medicine, Faculty of Medicine, University of Freiburg, Freiburg, Germany; 6 Department of Radiology, Faculty of Medicine, University of Freiburg, Freiburg, Germany; 7 Department of Neurology, Faculty of Medicine, University of Freiburg, Freiburg, Germany

**Keywords:** Intracerebral hemorrhage, ARDS, COVID-19

## Abstract

**Background:**

Hypercoagulability in Coronavirus Disease 2019 (COVID-19) causes deep vein thrombosis and pulmonary embolism necessitating systemic anticoagulation. Case reports of intracerebral hemorrhages in ventilated COVID-19 patients warrant precaution. It is unclear, however, if COVID-19 patients with acute respiratory distress syndrome (ARDS) with or without veno-venous extracorporeal membrane oxygenation therapy (VV-ECMO) have more intracerebral hemorrhages (ICH) compared to other ARDS patients.

**Methods:**

We conducted a retrospective observational single-center study enrolling all patients with ARDS from 01/2018 to 05/2020. PCR-positive SARS-CoV-2 patients with ARDS were allocated to the COVID-19 group. Propensity score matching was performed for age, VV-ECMO, and bleeding risk.

**Results:**

A total of 163 patients with moderate or severe ARDS were identified, 47 (28.8%) in the COVID-19 group, and 116 (71.2%) in the non-COVID-19 group. In 63/163 cases (38.7%), VV-ECMO therapy was required. The ICU survival was 52.8%. COVID-19 patients were older, more often male, and exhibited a lower SOFA score, but the groups showed similar rates of VV-ECMO therapy. Treatments with antiplatelet agents (*p* = 0.043) and therapeutic anticoagulation (*p* = 0.028) were significantly more frequent in the COVID-19 patients. ICH was detected in 22 patients (13.5%) with no statistical difference between the groups (11.2 vs. 19.1% without and with SARS-CoV-2, respectively, *p* = 0.21). Propensity score matching confirmed similar rates of ICH in both groups (12.8 vs. 19.1% without and with SARS-CoV-2, respectively, *p* = 0.57), thus leveling out possible confounders.

**Conclusions:**

Intracerebral hemorrhage was detected in every tenth patient with ARDS. Despite statistically higher rates of antiplatelet therapy and therapeutic anticoagulation in COVID-19 patients, we found a similar rate of ICH in patients with ARDS due to COVID-19 compared to other causes of ARDS.

**Supplementary Information:**

The online version contains supplementary material available at (doi:10.1007/s12028-021-01202-7).

## Introduction

Hypercoagulable states appear to be a challenging problem in Coronavirus Disease 2019 (COVID-19). It is probably caused by inflammatory changes similar to disseminated intravascular coagulopathy [1, 2]. Consequently, clinical and pathohistological reports about micro- and macro-thromboses as typical complications of COVID-19 in critically ill patients emphasize the need for anticoagulation [3–5]. Until today, however, there is no concrete evidence for managing anticoagulation beyond standard indications like atrial fibrillation [6, 7]. Ongoing studies focus on more aggressive anticoagulation to avoid thromboembolic complications (23 trials on prophylactic, intermediate, and therapeutic heparin doses register on clinicaltrials.gov as accessed on 06/17/2020). Higher anticoagulation aims are already targeted in some settings [8, 9].

Whereas pulmonary embolism and deep vein thrombosis have repeatedly been documented in hospitalized COVID-19 patients [3, 10], no study to date has examined the rate of major bleeding events. Only single-case reports [11, 12] of massive intracerebral hemorrhage (ICH) in COVID-19 and a case series of COVID-19 patients on veno-venous extracorporeal membrane oxygenation (VV-ECMO) with 4 out of 10 patients suffering from ICH have been published [13].

These raised the questions: Is it safe to intensify the anticoagulation in these patients? Might there be an increased (intracerebral) bleeding risk? Or are the hyperinflammation, impaired coagulation, and other bleeding risk factors comparable to a general population of patients with acute respiratory distress syndrome (ARDS)?

To illuminate this issue, we conducted a propensity score-matched study on the risk of intracerebral hemorrhage in patients with severe respiratory failure, comparing COVID-19 to non-COVID-19 ARDS.

## Methods

### Patient Selection

Patients with non-COVID-19 ARDS (ICD-10 code J80.01; J80.02; J80.03; J80.03; J80.09) were extracted from the hospital data system of the University Hospital of Freiburg, Germany, from 01/01/2018 to 05/31/2020 (non-COVID-19 group). Critically ill patients with PCR-confirmed SARS-CoV-2 infection and ARDS were enrolled from 03/2020 to 05/2020 (first admission 03/08/2020 of critically ill COVID-19 patient) and included in the COVID-19 group. We excluded patients younger than 18 years and with an intensive care unit (ICU) stay of less than 24 h. Also, patients with mild ARDS according to the Berlin definition were excluded. Both groups were followed until 06/13/2020. The study protocol of our retrospective monocentric study was approved by the local ethics committee (Ethik-Kommission der Albert-Ludwigs-Universität Freiburg im Breisgau, file number 333/20).

### Data Collection and Statistics

Clinical data reported in this study was obtained from our hospital data system and from documents of referring hospitals. We analyzed age, sex, sepsis-related organ failure assessment score (SOFA), length of stay, ICU survival, invasive mechanical ventilation (IMV), and extracorporeal therapies (renal replacement therapy, RRT, and VV-ECMO). The first documented value of D-Dimer levels during the stay was considered when no level at admission was available. Values of non-COVID-19 patients and COVID-19 patients were compared using Student’s *t*-test, Pearson´s Chi-square test, or Fisher´s exact test. Propensity score matching (1:1) was performed between the two groups matched for predetermined items including age, VV-ECMO treatment, and bleeding risk (HAS-BLED score) using SPSS (version 26, IBM, NYC, USA) and the optimal matching algorithm with a caliper of 0.1 was deployed. The HAS-Bled score is a well-validated risk score for major bleeding in patients taking anticoagulants because of atrial fibrillation [14, 15]. Results were considered statistically significant if the *p*-value was below 0.05. Graphs were designed with Prism (version 8 GraphPad, San Diego, USA).

### Patient Management

University Hospital of Freiburg is a tertiary treatment center for ARDS and ECMO-therapy with a high rate of hospital referrals for ECMO evaluation. Patients were transferred from the emergency department, from regular wards, or from primary and secondary treatment centers to medical and surgical ICUs per the local pandemic management protocol. ARDS was classified according to the Berlin classification [16]. If VV-ECMO was found necessary, ARDS was considered “severe”, since the calculation of the Horowitz index required for the Berlin classification is not possible in VV-ECMO.

All patients received ARDS treatment following current guidelines (including lung-protective ventilation, permissive hypercapnia, and therapeutic positioning maneuvers like pronation) as well as therapies required by the underlying cause of ARDS [7, 17]. During the recruitment period for COVID-19 patients in this study, steroids were not recommended by COVID-19 guidelines, and muscle relaxation was only rarely applied in early ARDS at our center.

If ARDS was not manageable with conservative strategies, VV-ECMO therapy was assessed by an interdisciplinary team including an intensivist, a perfusionist, and at least one registered nurse. VV-ECMO therapy was discouraged by the local guideline in cases of IMV > 7 days (without lung protection), preexisting acute intracerebral hemorrhage, uncontrolled cancer or coagulopathy, and in elderly patients (no cut-off). Standard VV-ECMO cannulation was performed with a dual-lumen jugular cannula (Avalon Elite™ Getinge Group, Rastatt, Germany), or bifemoral using two venous cannulas (Getinge Group, Rastatt, Germany). VV-ECMO therapies were conducted on the Stöckert Centrifugal Pump Console (SCPC) (LivaNova, Munich, Germany) or the Cardiohelp-System (Maquet, Rastatt, Germany). Sets were primed with 5000 IE of unfractionated heparin in 700 ml crystalloid solution.

Anticoagulation strategies followed in-house standard operating procedures and current guidelines for thromboprophylaxis [18]. Specifically, according to local standards, critically ill patients received a thromboprophylaxis of continuous unfractionated heparin (typically 2500 IU in 24 h hours IV), unless higher doses were required for the treatment of comorbidities. Non-COVID-19 VV-ECMO patients were managed with unfractionated heparin targeting an activated partial thromboplastin time (aPTT) of 40–50 s. Since 04/03/2020, COVID-19 patients on VV-ECMO were treated with a higher coagulation target of aPTT of 50–70 s in response to the COVID-19 hypercoagulability. In case of thrombotic events (pulmonary embolism, venous thrombosis, or VV-ECMO circuit clotting) during anticoagulation with unfractionated heparin, aPTT targets were adapted and a switch to Argatroban was considered.

### Intracerebral Hemorrhage

ICH was detected using cerebral computed tomography (CT) or magnetic resonance imaging (MRI). Cerebral imaging was done in patients suspected of a cerebral pathology or in cases of prolonged awakening. All CT scans and MRIs of the brain dated during ICU therapy or shortly after were used for analysis. An experienced radiologist and neurologist reviewed the scans in detail to describe the localization, extent, and pathogenesis of the bleeding. Preexisting microangiopathy was characterized utilizing the Fazekas classification [19] in cases without massive cerebral edema or massive ICH. In addition to hemorrhages, new ischemic events were also recorded. To define the overall bleeding risk, we assessed the HAS-BLED score on admission and 48 h prior to ICH.

## Results

### Baseline Characteristics

**Fig. 1:**
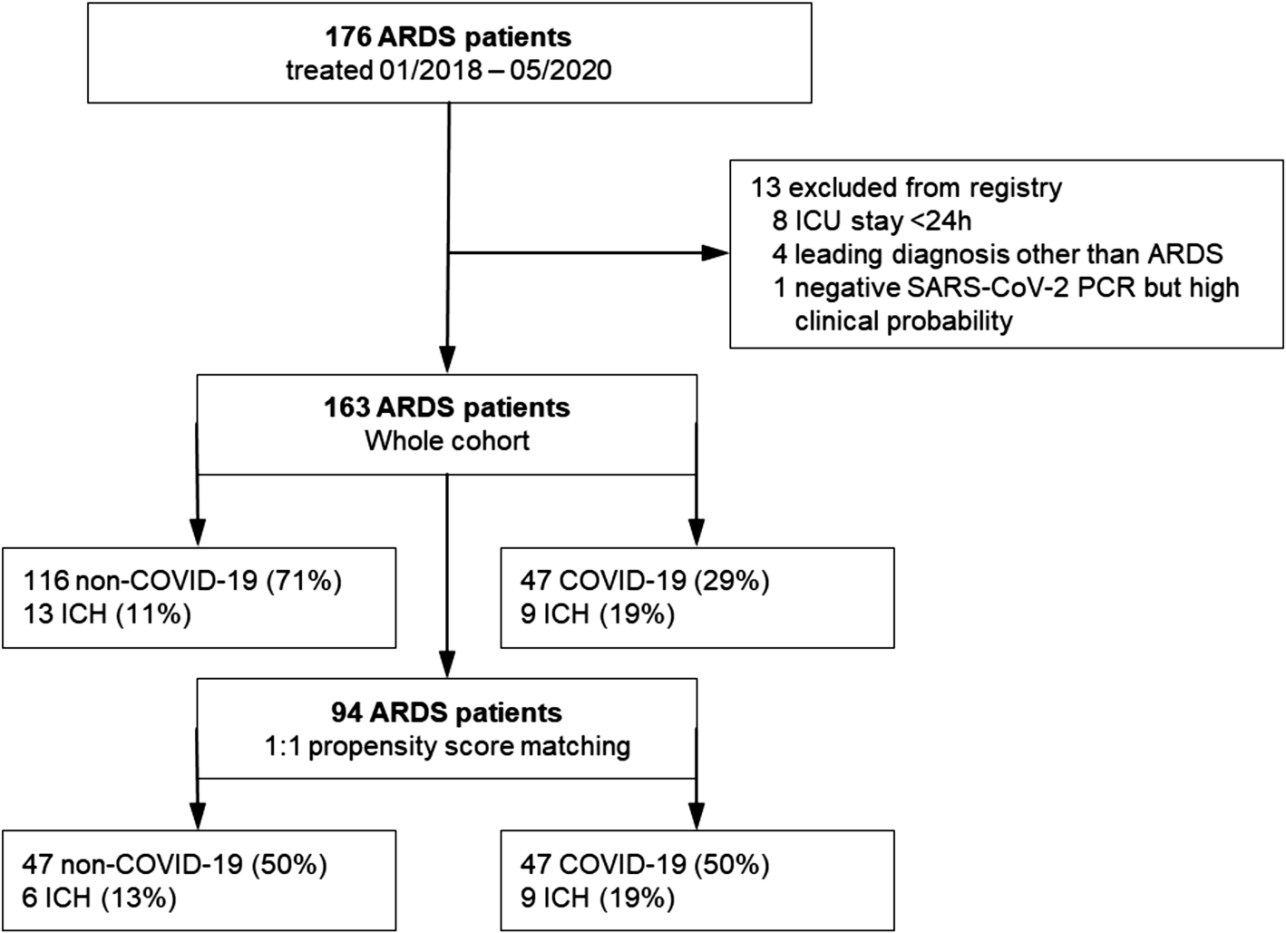
Flow chart of patients in registry. *ARDS* Acute respiratory distress syndrome; *ICH* Intracerebral hemorrhage

163 ARDS patients were included from January 2018 to May 2020 for analysis. The non-COVID-19 group consisted of 116 patients with ARDS. 70.0% had an infectious disease as the underlying cause, 13.8% resulted from non-infectious illnesses, and in 16.4% the cause remained obscure. Details are given in the electronic supplemental material (ESM) Table S4. Since March 2020, we included 47 critically ill COVID-19 ARDS patients (Table 1, Fig. 1). On average, patients were aged 60 ± 15 (24–92). There were more female patients in the non-COVID-19 group (42.4% vs. 19.1% in non-COVID-19 vs. COVID-19 respectively, *p* = 0.005). Non-COVID-19 patients were younger (58 ± 15 vs. 66 ± 13 in non-COVID-19 vs. COVID-19 respectively, *p* = 0.001) and exhibited higher SOFA scores (11 ± 4 vs. 9 ± 4 in non-COVID-19 vs. COVID-19 respectively, *p* = 0.006).

**Table 1:** Baseline characteristics of non-COVID-19 patients and COVID-19 patients

	All patients	COVID-19	Non-COVID-19	*p* value
Number of patients	163 (100%)	47 (28.8%)	116 (71.2%)	–
Age [years]	60 ± 15 (24–92)	66 ± 13 (31–92)	58 ± 15 (24–83)	**0.001**
Female gender	58 (35.6%)	9 (19.1%)	49 (42.2%)	**0.005**
Body mass index [kg/m^2^]	30 ± 10 (16–83)	28 ± 6 (18–51)	30 ± 11 (16–83)	0.229
Adipositas [BMI ≥ 30 kg/m^2^]	45 (31.5%)	13 (28.9%)	32 (33.0%)	0.653
Length of stay [days]	19 ± 17 (1–89)	23 ± 20 (1–89)	18 ± 16 (1–76)	0.750
SOFA Score	10 ± 4 (2–19)	9 ± 4 (2–17)	11 ± 4 (2–19)	**0.006**
ICU survival	86 (52.8%)	25 (53.2%)	61 (52.6%)	0.944
ARDS				
Mild	0	0	0	–
Moderate	53 (32.5%)	18 (38.3%)	35 (30.2%)	0.316
Severe	110 (67.5%)	29 (61.7%)	81 (69.8%)	0.316
paO2/FiO2 (on day 1)	111 ± 38 (35–227)	113 ± 38 (35–227)	110 ± 39 (36–222)	0.727
Highest PEEP (on day 1)	12 ± 4 (5–20)	12 ± 4 (5–19)	13 ± 4 (5–20)	0.360
Representative FiO2 at highest PEEP (on day 1)	60 ± 18 (30–100)	65 ± 19 (40–100)	58 ± 17 (30–100)	**0.023**
Invasive mechanical ventilation (IMV)	146 (89.6%)	40 (85.1%)	106 (91.4%)	0.235
Duration of IMV [days]	20 ± 21 (1–129)	23 ± 22 (1–89)	19 ± 20 (1–129)	0.349
Renal replacement therapy	42 (25.8%)	13 (27.7%)	29 (25.0%)	0.725
Veno-venous extracorporeal membrane oxygenation (VV-ECMO)	63 (38.7%)	14 (29.8%)	49 (42.2%)	0.139
Duration of VV-ECMO [days]	17 ± 18 (2–72)	22 ± 20 (2–71)	16 ± 17 (2–72)	0.272

A *p*-value below 0.05 is highlighted in bold

Baseline characteristics are displayed for all patients, in patients with COVID-19 ARDS and non-COVID-19 ARDS. Data are n (%) or mean with standard deviation and range. Student´s *t*-test, Pearson´s Chi-square, or Fisher´s exact test was performed to derive *p*-values

*ARDS* Acute respiratory distress syndrome, *ICU* Intensive care unit,* IMV* Invasive mechanical ventilation, *PEEP* Positive end-expiratory pressure, *SOFA score* Sepsis-related organ failure assessment score, * VV*-*ECMO* Veno-venous extracorporeal membrane oxygenation

A total of 63/163 (38.7%) patients underwent VV-ECMO therapy. The rate of VV-ECMO therapy was similar between the groups. Furthermore, the length of ICU stay, days on IMV, and days on VV-ECMO were similar. ICU survival in the whole group was 52.8% which was comparable (52.6% vs. 53.2% in non-COVID-19 vs. COVID-19, *p* = 0.944). Details on the mode of death are given in the ESM (Table S3).

### Risk Factors for Bleeding and Anticoagulation

Patients in the whole cohort presented with a low HAS-BLED score of 1.6 ± 1.3 (0–4), which was similar between the two groups (Table 2). The HAS-BLED score 48 h prior to ICH did not differ between non-COVID-19 and COVID-19 patients. Alcohol abuse (a risk factor in the HAS-BLED score) was detected significantly more often in the non-COVID-19 patients (*p* = 0.006). Antiplatelet therapy with one agent and dual antiplatelet therapy were less frequent in the non-COVID-19 patients.

**Table 2:** Risk factors for bleeding

	All patients( *N* = 163)	COVID-19 (*N* = 47)	Non-COVID-19 (N = 116)	*p* value
HAS-BLED score (on admission)	1.6 ± 1.3 (0–4)	1.7 ± 1.3 (0–4)	1.5 ± 1.3 (0–4)	0.382
Hypertension	74 (45.4%)	26 (55.3%)	48 (41.4%)	0.105
Abnormal liver function	15 (9.2%)	0	15 (15.5%)	**0.006**
Abnormal kidney function	27 (16.6%)	9 (19.1%)	18 (15.5%)	0.643
Former stroke	3 (1.8%)	2 (4.3%)	1 (0.9%)	0.200
Former tendency to bleed	2 (1.2%)	0	2 (1.7%)	0.365
Unstable INR values	0	0	0	–
Age > 65 years	71 (43.6%)	26 (55.3%)	45 (38.8%)	0.054
Blood thinner	47 (28.8%)	17 (36.2%)	30 (25.9%)	0.188
Alcohol abuse	16 (9.8%)	0	16 (13.8%)	**0.006**
Coagulopathy in patient´s history	2 (1.2%)	0	2 (1.7%)	0.365
indication for oral anticoagulation in patient´s history	33 (20.2%)	9 (19.1%)	24 (20.7%)	0.825
Any oral anticoagulation^a^	30 (18.4%)	10 (21.3%)	20 (17.2%)	0.547
Aspirin	22 (13.5%)	9 (19.1%)	13 (11.2%)	0.208
Any antiplatelet therapy	29 (17.8%)	13 (27.7%)	16 (13.8%)	**0.043**
Any dual antiplatelet therapy	6 (3.7%)	3 (6.4%)	3 (2.6%)	0.356
aPTT target				
< 40 s (thromboprophylaxis)	65 (39.9%)	14 (29.8%)	51 (44.0%)	0.094
40–50 s	23 (14.1%)	4 (8.5%)	19 (16.4%)	0.224
50–70 s	15 (9.2%)	8 (17.0%)	7 (6.0%)	**0.037**
60–80 s (therapeutic)	60 (36.8%)	21 (44.7%)	39 (33.6%)	**0.028**
Unfractionated heparin^b^	158 (96.9%)	43 (91.5%)	115 (99.1%)	**0.010**
Argatroban	19 (11.7%)	12 (25.5%)	7 (6.0%)	**0.001**
Platelets				
Platelets on admission (Tsd/µl)	217 ± 153 (17–913)	234 ± 111 (39–495)	210 ± 167 (17–913)	0.379
> 150 (Tsd/µl)	100 (61.3%)	35 (74.5%)	65 (56.0%)	**0.029**
149–100 (Tsd/µl)	24 (14.7%)	8 (17%)	16 (13.8%)	0.629
99–50 (Tsd/µl)	20 (12.3%)	2 (4.3%)	18 (15.5%)	0.063
< 50 (Tsd/µl)	19 (11.7%)	2 (4.3%)	17 (14.7%)	0.065
Platelets (Tsd/µl) 48 h prior to ICH	126 ± 93 (31–445)	161 ± 128 (36–445)	102 ± 51 (31–197)	0.217
Lowest platelets (Tsd/µl) if no ICH	126 ± 101 (1–478)	156 ± 90 (40–458)	115 ± 103 (1–478)	**0.031**

A *p*-value below 0.05 is highlighted in bold

Risk factors for bleeding are displayed for all patients, in patients with COVID-19 ARDS and non-COVID-19 ARDS. Data are *n* (%) or mean with standard deviation and range. Student´s *t*-test, Pearson´s Chi-square, or Fisher´s exact test was performed to derive *p*-values

*aPTT* Activated partial thromboplastin time, *HAS-BLED score* validated score for bleeding risk, *ICH* Intracerebral hemorrhage, *INR* International normalized ratio

^a^One patient received fibrinolysis and was not included in this group

^b^158/163 Unfractionated heparin, 18 were switched to Argatroban, 1 patient directly received Argatroban. 4 patients received only low molecular weight heparin

The initial platelet count was above 150 Tsd/µl, in 74.5% of COVID-19 patients compared to 56.0% of non-COVID-19 (*p* = 0.029). Very low initial counts (< 50 Tsd/µl) tended to occur more often in the non-COVID-19 group (*p* = 0.065).

Following local guidelines, anticoagulation targets were higher in COVID-19 patients. Significantly more COVID-19 patients were given therapeutic anticoagulation (with an aPTT of 60–80 s) than non-COVID-19 patients (33.6% vs. 44.7% in non-COVID-19 vs. COVID-19, *p* = 0.028). On admission, laboratory tests for coagulation, including total platelet count, INR, and aPTT, were similar between the groups (Table S2). Unfractionated heparin was significantly more often used in non-COVID-19 patients (*p* = 0.01), and more COVID-19 patients were switched to Argatroban (*p* = 0.001). Blood pressure excess (documented systolic blood pressure > 180 mmHg > 30 min. 48 h prior to ICH) or aPTT excess (aPTT greater than 80 s 48 h prior to ICH) were rare (Table 3). The Fazekas scores between the groups were comparable (1.3 ± 0.9 vs. 1.0 ± 0.8 in non-COVID-19 vs. COVID-19, *p* = 0.293) (Table S1).

**Table 3:** Number and characteristics of intracerebral hemorrhage (ICH)

	All patients (*N* = 163)	COVID-19 (*N* = 47)	Non-COVID-19 (*N* = 116)
Rate of cerebral imaging^a^	96 (58.3%)	26 (55.3%)	70 (60.3%)
Intracerebral hemorrhage (ICH)	22 (13.5%)	9 (19.1%)	13 (11.2%)
Fatal intracerebral hemorrhage	6 (3.7%)	3 (6.3%)	3 (2.5%)
Characteristics of ICH	All ICH(*N* = 22)	COVID-19 ICH(*N* = 9)	Non-COVID-19 ICH(*N* = 13)
Typical localization of ICH only (basal ganglia, brainstem, cerebellum)	1 (4.5%)	0	1 (7.7%)
Atypical localization of ICH only	9 (40.9%)	2 (22.2%)	7 (61.5%)
Cortical	2 (9.1%)	1 (11.1%)	1 (7.7%)
Subcortical	7 (31.8%)	1 (11.1%)	6 (46.2%)
SAH only	5 (22.7%)	2 (22.2%)	3 (23.1%)
SDH/EDH only	3 (13.6%)	1 (11.1%)	2 (15.4%)
Multiple (types of bleeding)	4 (18.2%)	4 (44.4%)^b^	0
Fluid level observed	3 (13.6%)	3 (33.3%)^c^	0
Additional intraventricular hemorrhage	6 (27.3%)	2 (22.2%)	4 (30.7%)
RASS on day of hemorrhage	− 3.2 ± 1.8 (− 5–0)	− 3.1 ± 2.2 (− 5–0)	− 3.4 ± 1.6 (− 5–0)
Length of stay until hemorrhage	17.6 ± 17.6 (0–63)	20.4 ± 14.8 (2–47)	15.5 ± 19.6 (0–63)
Hemorrhage during IMV^d^	19 (86.4%)	7 (77.8%)	12 (92.3%)
Hemorrhage during RRT	8 (36.4%)	3 (33.3%)	5 (38.5%)
Hemorrhage during VV-ECMO	13 (50.0%)	4 (44.4%)^e^	9 (69.2%)
HAS-BLED Score 48 h prior to hemorrhage	2.3 ± 1.3 (0–4)	2.3 ± 1.4 (0–4)	2.3 ± 1.3 (0–4)
Blood pressure excess 48 h prior to hemorrhage	5 (27.3%)	2 (22.2%)	3 (23.1%)
aPTT excess 48 h prior to hemorrhage	4 (18.0%)	1 (11.1%)	3 (23.1%)

Characteristics of intracerebral hemorrhage are displayed for all patients, in patients with COVID-19 ARDS and non-COVID-19 ARDS. Data are n (%) or mean with standard deviation and range. No statistical significance could be found in rate of cerebral imaging (*p* = 0.555), intracerebral hemorrhage (*p* = 0.208) or fatal intracerebral hemorrhage (*p* = 0.356) between COVID-19 and non-COVID-19 patients

*aPTT* Activated partial thromboplastin time, *EDH* Epidural hemorrhage, *HAS*-*BLED* Validated score for bleeding risk, *ICH* Intracerebral hemorrhage,* IMV* Invasive mechanical ventilation, *SAH* subarachnoid hemorrhage, *SDH* subdural hematoma, *RASS* Richmond agitation sedation scale, *RRT* Renal replacement therapy, *VV*-*ECMO* Veno-venous extracorporeal membrane oxygenation

^a^94 Cerebral computed tomographies (CT) only, 2 magnetic resonance imaging (MRI) of the brain only, 6 CT and MRI

^b^2 Patients with subcortical ICH + SAH (22.2%); 1 patient with subcortical ICH + SDH + SAH (11.1%); 1 patient with subcortical + cortical ICH 1 (11.1%)

^c^Only observed in multiple

^d^No ICH under non-invasive ventilation or nasal high flow

^e^In one patient ICH was detected after VV-ECMO weaning but associated with VV-ECMO therapy and included in this group

### Intracerebral Hemorrhage

**Fig. 2:**
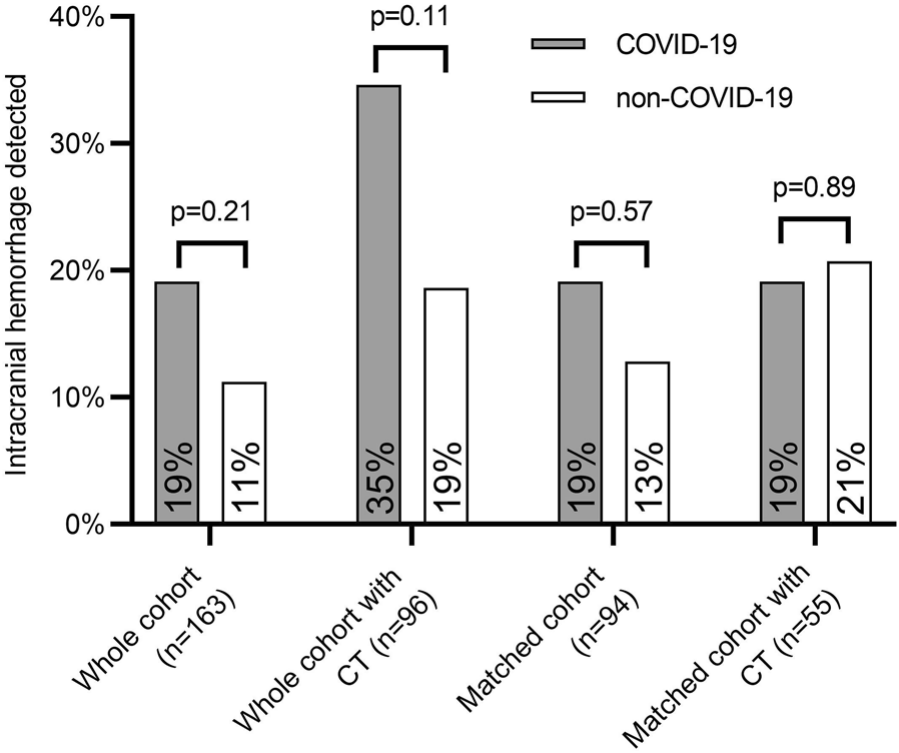
Intracerebral hemorrhage in acute respiratory distress syndrome: There was no significant difference between patients with and without COVID-19 in respect to intracerebral hemorrhage as diagnosed by cerebral computed tomography (CT)

In 96/163 (58.3%) patients, cerebral imaging was performed (Table 3). Cerebral scans were conducted at similar rates in non-COVID-19 and COVID-19 patients (60.3% vs. 55.3%, respectively, *p* = 0.555). In total, 13 patients were diagnosed with ICH in the non-COVID-19 group, compared to 9 patients in the COVID-19 group (see supplemental Fig. 1 for representative examples). We found no statistically significant difference between the two groups evaluating any ICH or fatal ICH in the unmatched cohort (Table 3). After propensity score matching, similar rates of ICH were found (Fig. 2).

One non-COVID-19 patient had a typical bleeding localization for hypertensive ICH (including basal ganglia, upper brainstem, or cerebellum). All other intraparenchymal ICH showed atypical cortical or subcortical localization without difference between the groups (7/13 vs. 2/9 in non-COVID-19 vs. COVID-19, *p* = 0.203). However, a combination of multiple types of intracranial bleeding in a single patient was only seen in the COVID-19 group (0/13 vs. 4/9 in non-COVID-19 vs. COVID-19, *p* = 0.017). Fluid levels within ICH were exclusively ascertained in COVID-19 patients (0/13 vs. 3/9 in non-COVID-19 vs. COVID-19, p = 0.055) (for details see Table 3).

### Inflammation and Thrombotic Events

Markers of inflammation were assessed on admission in the form of white blood cell count, C-reactive protein (CRP), and procalcitonin (PCT) (Table S2). PCT tended to be higher in the non-COVID-19 group (16.0 ± 52.5 vs. 1.2 ± 3.1 in non-COVID-19 vs. COVID-19, *p* = 0.672). No differences were detected between the two groups in white blood cell count and CRP on admission.

Sub-group analysis of COVID-19 patients with and without ICH did not reveal statistically significant differences in the inflammation markers on admission. Specifically, white blood cell count (8.9 ± 5.2 vs. 7.7 ± 4.4 Tsd/µl in COVID-19 with ICH vs. COVID-19 without ICH, p = 0.049), CRP (145 ± 67 vs. 150 ± 107 mg/dl, respectively, p = 0.896), PCT (1.0 ± 1.2 vs. 1.3 ± 3.4 ng/ml, respectively, p = 0.802), and interleukin 6 (2752 ± 6562 vs. 502 ± 732 pg/ml, respectively, *p* = 0.334) were similar.

To investigate markers for hypercoagulability, D-Dimers, events of ischemic stroke, pulmonary embolism, and clotting in the extracorporeal systems were assessed (Table S1). Initial D-Dimer levels trended higher in non-COVID-19 patients (*p* = 0.346). Interestingly, there was a trend towards a higher rate of newly diagnosed stroke in the non-COVID-19 group (12.9% vs. 6.4% for non-COVID-19 and COVID-19 group, respectively, *p* = 0.144). In the whole cohort, the majority (77.8%) of strokes were of embolic origin without proximal vessel occlusion. COVID-19 patients suffered significantly more often from pulmonary embolism (7.8% vs. 21.3%, non-COVID-19 vs. COVID-19, *p* = 0.004). Clotting in extracorporeal circuits (VV-ECMO, RRT) was more frequent in the COVID-19 group (VV-ECMO clotting: 17.3 vs. 23.4% in non-COVID-19 vs. COVID-19, *p* = 0.013; clotting in RRT: 5.2 vs. 12.6% in non-COVID-19 vs. COVID-19, *p* = 0.091).

## Discussion

To our best knowledge, this is the first study elucidating the risk of intracerebral hemorrhage in COVID-19 ARDS. In the current work, ICH was found in 13.5% of the ARDS patients treated at a tertiary ECMO center. Numerically the ICH rate was higher in COVID-19 patients but did not reach statistical significance either in the whole cohort or after propensity score matching.

The rate of ICH in our ARDS patients (conservative or on VV-ECMO) is in line with a non-COVID-19 ARDS cohort from the UK at a comparable structured ECMO center (ICH detected in the whole cohort 14.0%; 48/342). Comparing subgroups, our rate of 20.1% in VV-ECMO and 7.6% in patients without VV-ECMO is similar to the published results (16.4% and 9.0% for patients with and without VV-ECMO, respectively) [20].

A far lower rate of ICH has been reported by the ELSO registry in non-COVID ARDS patients on VV-ECMO (3.6%; 181/4,988) and in patients enrolled in the EOLIA study (2.4%; 3/124) [21, 22].

The high ICH rate found in our cohort might reflect the severity of the illness requiring extracorporeal circuits and anticoagulant therapy in a relevant portion of patients. The majority of our patients were managed with anticoagulant therapy more intense than thromboprophylaxis. Initial low platelet counts in the non-COVID-19 patients might be a result of a higher number of hemato-oncological preconditions and patients with known liver dysfunction. Intracerebral bleeding risk in the COVID-19 patients was possibly driven by age, male gender, and preexisting antiplatelet therapy.

Furthermore, ICU survival in our cohort (52.8%) is lower than reported by other data for severe ARDS (62.0%) [23]. Our registry contains predominantly severely ill patients as suggested by the predicted mortality of 50.0% according to the SOFA score [24], which might explain the survival difference.

Since the rate of ICH detected in COVID-19 seems comparable to other ARDS, our findings might suggest a general pathomechanism of ICH in ARDS, defined by cerebral damage due to systemic inflammation, accompanied by extracorporeal circuits, and patient inherent factors, each facilitating bleeding.

Some phenotypes of pulmonary failure proceed with a hyperinflammatory immune response, circulatory failure, and subsequent multi-organ failure. The underlying hypercoagulability results in clinical complications pronounced in COVID-19 ARDS, such as deep vein thrombosis, pulmonary artery thrombosis, or clotting during extracorporeal organ replacement therapies [10, 25]. The detection of a high rate of new strokes (11.0%) in the cohort, mainly due to embolic events, might also be an expression of inflammation-induced hypercoagulability.

We can only indirectly measure inflammation and hypercoagulability through elevated blood markers for inflammation (like CRP, interleukin 6, ferritin) along with elevated D-Dimers, prolonged prothrombin time, and thrombocytopenia [26–28]. A local reaction such as a pathogen-associated endotheliitis or a systemic inflammation might also trigger these prothrombotic events [2].

In SARS-CoV-2, neuroinvasion and neurotropism are suspected due to the neurotropic and neuroinvasive nature of the coronavirus in general. This is reflected in the increasing number of patients with SARS-CoV-2 suffering from neurological manifestations [29]. SARS-CoV-2 viral protein has been isolated in brain stem cells, and brainstem neuroinflammation has been detected in a post-mortem analysis. However, Matschke et al. did not find correlated central nervous system damage [30].

Due to the retrospective nature of our study, we cannot determine if ICH in ARDS is caused by a primary hemorrhage or an embolic event with secondary hemorrhage. Various forms of ICH were detected in the COVID-19 cohort. The thorough review of the CTs did not show a unique entity or a patterned timing of bleeding events.

Despite statistically higher rates of antiplatelet therapy and therapeutic anticoagulation in COVID-19 patients compared to non-COVID-19 patients in this study, the resulting rates of ICH were the same. Therefore, a change to standard of care cannot be derived from our retrospective data. As usual, individual risks for bleeding and therapy associated factors should be taken into account. The substantial rate of ICH in both, COVID-19 and non-COVID-19 ARDS patients, seen in this registry is alarming and should be investigated in further trials.

## Limitations

Limitations of the present study naturally include the small patient number. Moreover, as a tertiary treatment center, our patients were biased with moderate or severe ARDS as well as high SOFA scores. The rate of ICH in our cohort with 13.5% (22/163) and the rate for strokes of 11.0% (18/163) might not be representative for cohorts in primary and secondary treatment centers with lower SOFA scores and lower rates of organ replacement therapies. Furthermore, CT scans were performed based on clinical judgment. It remains unclear how many clinically silent events occurred. Also, our anticoagulation regimes under VV-ECMO therapy are empiric for both COVID-19 and non-COVID-19 patients and tended to be more aggressive for COVID-19 patients. Moreover, the COVID-19 group had more male patients overall, with male gender being a known factor for ICH [31]. The patients in the overall cohort were older in the COVID-19 group, hence why propensity score matching was performed to overcome a possible bias. In our cohort, we did detect similar bleeding rates in females and males with a tendency towards more bleeding in female patients (19.0% vs. 10.5% for females and males, respectively, *p* = 0.153). Finally, the HAS-BLED score was not designed to predict bleeding risk in VV-ECMO patients with ARDS and therefore might not accurately correlate with bleeding in this cohort.

## Conclusions

Intracerebral hemorrhage was detectable in one out of every ten patients with ARDS. Despite statistically higher rates of antiplatelet therapy and therapeutic anticoagulation in COVID-19, we found a similar rate of ICH in patients with ARDS due to COVID-19 compared to other causes of ARDS.

## Supplementary Information

Supplementary material (2377 kb)

## Data Availability

The datasets analyzed during the current study are available from the corresponding author on reasonable request.

## Abbreviations

aPTT: Activated partial thromboplastin time
ARDS: Acute respiratory distress syndrome
BMI: Body mass index
CT: Computed tomography
COVID-19: Coronavirus disease 2019
EDH: Epidural hemorrhage
ESM: Electronic supplemental material
HAS-BLED Score: Validated score for bleeding risk
ICH: Intracerebral hemorrhage
ICU: Intensive care unit
IMV: Invasive mechanical ventilation
MRI: Magnetic resonance imaging
PEEP: Positive end-expiratory pressure
RASS: Richmond Agitation Sedation Scale
RRT: Renal replacement therapy
SAH: Subarachnoid hemorrhage
SARS-CoV-2: Severe acute respiratory coronavirus 2
SDH: Subdural hematoma
SOFA Score: Sepsis-related organ failure assessment score
VV-ECMO: Veno-venous extracorporeal membrane oxygenation

## Permissions Information

The authors do hereby declare that all illustrations and figures in the manuscript are entirely original and do not require reprint permission.
