# Supplementary material for: Intracerebral Hemorrhage in COVID-19 Patients with Pulmonary Failure: A Propensity Score-Matched Registry Study
Source: Neurocrit Care. 2021 Feb 23;34(3):739–47. doi: 10.1007/s12028-021-01202-7 (PMC7899797; doi:10.1007/s12028-021-01202-7)
Supplement: Supplementary file 1 — Supplementary material (2377 kb) [file 12028_2021_1202_MOESM1_ESM.docx]

**Supplemental Table 1: stroke, microangiopathy and clotting events**

|  | all patients  (N=163) | COVID-19  (N=47) | non-COVID-19  (N=116) | *p*-value |
| --- | --- | --- | --- | --- |
| new stroke^a^ | 18 (11.0%) | 3 (6.4%) | 15 (12.9%) | 0.144 |
| embolic | 14 | 2 | 12 |  |
| hemodynamic | 1 | 0 | 1 |  |
| microangiopathic | 3 | 1 | 2 |  |
| proximal vessel occlusion | 0 | 0 | 0 |  |
| width of 3^rd^ ventricle^b^ | 7.6±2.6 (2.9-15.7) | 9.1±2.5 (4.4-15.7) | 7.1±2.4 (2.9-12.1) | 0.274 |
| microangiopathy (Fazekas)^c^ | 1.2±0.9 (0-4) | 1.0±0.8 (0-3) | 1.3±0.9 (0-4) | 0.289 |
| 0 | 19 | 7 | 12 |  |
| 1 | 45 | 12 | 33 |  |
| 2 | 18 | 6 | 12 |  |
| 3 | 8 | 1 | 7 |  |
| 4 | 1 | 0 | 1 |  |
| pulmonary embolism (CT)^d^ | 19 (11.7%) | 10 (21.3%) | 9 (7.8%) | 0.004 |
| VV-ECMO Clotting^e^ | 31 (19.0%) | 11 (23.4%) | 20 (17.2%) | 0.013 |
| RRT Clotting^e^ | 12 (7.4%) | 6 (12.6%) | 6 (5.2%) | 0.091 |

New strokes, their pathogenesis and clotting events are displayed for all patients, in patients with COVID-19 ARDS and non-COVID-19 ARDS. Data are n (%) or mean with standard deviation and range. Student´s *t*-test, Pearson´s chi-square, or Fisher´s exact test was performed to derive *p*-values. CT, computed tomography; VV-ECMO, veno-venous extracorporeal membrane oxygenation; RRT, renal replacement therapy. ^a^ 94 cerebral computed tomographies (CT) only, 2 magnetic resonance imaging (MRI) of the brain only, 6 CT and MRI. ^b^ 6 patients could not be assessed according to the width of the 3^rd^ ventricle either because of cerebral edema or massive intracerebral hemorrhage ^c^ 5 patients could not be assessed according to the Fazekas classification either because of cerebral edema or massive intracerebral hemorrhage ^d^ Pulmonary embolism confirmed in CT pulmonary angiogram ^e^ Clotting in the ECMO system or clotting in renal replacement systems which acquired (partial) system exchange are presented.

**Supplemental Table 2: laboratory measure**

|  | all patients  (N=163) | COVID-19  (N=47) | non-COVID-19  (N=116) | *p*-value |
| --- | --- | --- | --- | --- |
| on admission |  |  |  |  |
| white blood counts (Tsd/µl) | 14.5±11.9 (0.6-99.0) | 10.5±5.1 (3.0-29.3) | 16.2±13.3 (0.6-99.0) | 0.313 |
| hemoglobin (g/dl) | 11.0±2.4 (6.0-16.0) | 12.6±2.1 (7.0-15.6) | 10.4±2.3 (6.0-16.1) | 0.102 |
| platelets (Tsd/µl) | 217±153 (17-913) | 234±111 (39-495) | 210±167 (17-913) | 0.379 |
| INR | 1.3±0.5 (0.9-4.8) | 1.2±0.5 (0.9-4.0) | 1.3±0.5 (0.9-4.8) | 0.576 |
| aPTT (s) | 46±24 (22-140) | 41±18 (26-140) | 49±26 (22-140) | 0.824 |
| C-reactive protein (mg/dl) | 174±114 (3-541) | 149±100 (8-503) | 185±118 (3-541) | 0.782 |
| procalcitonin (ng/ml) | 11.64±44.72 (0.1-400) | 1.21±3.07 (0.1-19.5) | 15.97±52.53 (0.1-400) | 0.672 |
| creatinine (mg/dl) | 1.52±1.03 (0.35-6.18) | 1.44±0.91 (0.52-4.70) | 1.56±1.08 (0.35-6.18) | 0.921 |
| GFR | 63±34 (8-145) | 62±30 (11-111) | 63±35 (8-145) | 0.819 |
| aspartate aminotransferase  (U/l) | 165±563 (12-5546) | 81±85 (20-515) | 200±667 (12-5546) | 0.539 |
| alanine aminotransferase  (U/l) | 83±305 (7-3724) | 54±54 (9-283) | 95±362 (7-3724) | 0.588 |
| (first) d-dimer (mg/l) | 9.7±10.5 (0.2-35.2) | 5.9±8.2 (0.2-35.2) | 13.0±11.1 (0.3-35.2) | 0.346 |

Laboratory measures are displayed for all patients, in patients with COVID-19 ARDS and non-COVID-19 ARDS. Data are mean with standard deviation and range. Student´s *t*-test, Pearson´s chi-square, or Fisher´s exact test was performed to derive *p*-values. aPTT, activated partial thromboplastin time; GFR, glomerular filtration rate.

**Supplemental Table 3: cause of death**

|  | all non-survivors  (N=77) | COVID-19 non-survivors  (N=22) | non-COVID-19 non-survivors  (N=55) |
| --- | --- | --- | --- |
| cause of death |  |  |  |
| refractory ARDS | 30 (39.0%) | 4 (18.2%) | 26 (47.3%) |
| refractory multi-organ failure | 31 (40.3%) | 11 (50.0%) | 20 (36.4%) |
| withdrawal because of DNR | 7 (9.1%) | 4 (18.2%) | 3 (5.5%) |
| intracerebral hemorrhage | 6 (7.8%) | 3 (13.6%) | 3 (5.5%) |

Cause of death is displayed for all patients, in patients with COVID-19 ARDS and non-COVID-19 ARDS. Data are n (%). ARDS, acute respiratory distress syndrome; DNR, do not resuscitate order.

**Supplemental Table 4: cause of ARDS in non-COVID ARDS**

|  | non-COVID-19  (N=116) |
| --- | --- |
| influenza | 29 (25%) |
| other virus (except SARS-CoV-2) | 8 (6.9%) |
| *Streptococcus pneumonia* | 5 (4.3%) |
| other bacterium | 29 (25%) |
| *Pneumocystis jirovecii* | 10 (8.6%) |
| *non-infectious* | 16 (13.8%) |
| *obscure* | 19 (16.4%) |

Cause of ARDS is displayed for non-COVID ARDS. Data are n (%). ARDS, acute respiratory distress syndrome.

**Supplemental Figure 1**

Figure 1: cerebral computed tomographies of three intracerebral hemorrhages in COVID-19 patients

The cerebral computed tomographies show three cases of fatal intracerebral hemorrhages in COVID-19 patients with severe pulmonary failure.

Case 1: 60-year-old female obese health-care worker undergoing steroid therapy due to fibromyalgia. SARS-CoV-2 pneumonia resulted in severe ARDS on VV-ECMO and RRT. On day 19 of her clinical course and day 10 after VV-ECMO implantation, she presented with anisocoric pupils. The intracerebral hemorrhage was judged fatal.

Case 2: 49-year-old otherwise healthy male with severe ARDS on VV-ECMO after SARS-CoV-2 transmission presumably at his family doctor´s practice. On day 7 after hospitalization and on day 3 after VV-ECMO implantation a fatal intraparenchymatoes hemorrhage occurred. Liquor remained negative in the virological testing according to SARS-CoV-2.

Case 3: 69-year-old male with COVID-19 ARDS. Patient´s history comprised coronary artery disease and atrial fibrillation. On day 9, he presented with anisocoric pupils and the CT revealed a right frontal cerebral bleeding. Evacuation of the bleeding was performed but the patient deceased. Neuropathology work-up showed a fresh parenchymal and subarachnoidal hemorrhage and reactive gliotic tissue.
